# SEVUparin as a potential Adjunctive Treatment in children with severe malaria: A phase I trial safety and dose finding trial (SEVUSMAART)

**DOI:** 10.12688/wellcomeopenres.20111.1

**Published:** 2023-10-20

**Authors:** Kathryn Maitland, Mainga Hamaluba, Nchafatso Obonyo, Emmanuel Oguda, Christabel Mogoka, Thomas N. Williams, Mike Chaponda, Sam Miti, Luc Kambale Kamavu, Roisin Connon, Diana M. Gibb, Arjen Dondorp, Nick Day, Nick White, A. Sarah Walker, Elizabeth C. George, Severe Malaria in African Children A Research and Trials (SMAART) consortium

**Affiliations:** 1 Department of Infectious Disease and Institute of Global Health and Innovation, Imperial College London, London, England, UK; 2 Clinical Research, 1. KEMRI-Wellcome Trust Research Programme, Kilifi, Kilifi, Po Box 230, Kenya; 3 Centre of Ndola Teaching Hospital, 3. Tropical Diseases Research, Ndola, P.O Box 71769, Zambia; 4 Paediatrics, Nchelenge Hospital, Nchelenge, Luapula Province, Zambia; 5 Paediatric, Arthur Davison Children's Hospital, Ndola, P.O. Box 240227, Zambia; 6 Medical Research Council Clinical Trials, University College London, London, England, WC1V 6LJ, UK; 7 Clinical Trials, Mahidol Oxford Tropical Medicine Research Unit, Bangkok, 10400, Thailand

**Keywords:** severe malaria, adjunctive therapy, children, Africa, clinical trial, heparin-like molecule

## Abstract

**Background:**

Even on the best antimalarial treatments (injectable artesunate) African children with severe malaria have poor outcomes with most deaths occurring early in the course of hospital admission (<24hours). Lactic acidosis, largely due to impairment of the microcirculatory flow due to parasite sequestration, is a main risk factor for poor outcome. There are no adjuvant treatments for severe malaria that target this complication. Sevuparin, a heparin-like drug, binds to *Plasmodium falciparum* erythrocyte membrane protein blocking merozoite invasion, preventing cytoadherence and transiently de-sequestering infected erythrocytes. Leading to improved microcirculatory flow by reversing/preventing parasite sequestration. If given early during admission this could result in improvements in outcomes . Sevuparin has been shown to be safe and well tolerated in adults with only some mild transient effects on activated partial thromboplastin time (APTT) were reported, without clinical consequences.

**Methods:**

A Phase I trial designed to provide data on safety, dosing, feasibility of sevuparin as an adjuvant therapy in Kenya and Zambian children with severe malaria complicated by lactic acidosis (> 2mmol/l). Three intravenous doses will be given at admission (0 hours), 8 and 16 hours. APPT will be measured 1 hour after each dose (to assess maximum toxicity). Studying 20 children will allow sufficient data on safety to be generated across a range of doses to identify the maximum tolerated dose (MTD) using the Continual Reassessment Method, which adapts or informs subsequent doses for each child based on the data from previously enrolled children. The MTD will be identified based on the dose-toxicity model updated by each previous patient’s APTT results using standard methods.

**Conclusions:**

The results of the Phase I trial will identify the final dose to be tested in a Phase II trial in terms of both efficacy and safety outcomes.

**Registration:**

PACTR number: 202007890194806 (date 20/07/2020) ISRCTN32271864 (date 28/07/2021)

## Abbreviations

ACT              Artemisinin combination therapy

AE                Adverse event

APTT           Activated partial thromboplastin time

AT                Antithrombin

BCS              Blantyre Coma Scale

CI                Confidence interval

CM              Cerebral Malaria

CRF             Case Record Form

CRM            Continuous Reassessment Method

CTC             Common Toxicity Criteria

DIC              Disseminated intravascular coagulation

DLT             Dose limiting toxicity

DMC           Data Monitoring Committee

ECG            Electrocardiogram

ED50           Median effective dose (effective dose for 50% of people receiving the drug)

GMP            Good Manufacturing Practice

HDU            High Dependency Unit

HRP2 *P. falciparum* Histidine Rich Protein 2

IV                 Intravenous

KCH              Kilifi County Hospital

KEMRI         Kenya Medical Research Institute

KWTRP        Kilifi Wellcome Trust Research Programme

ICREC          Imperial College Research Ethics Committee

MRC             CTU Medical Research Council Clinical Trials Unit

MTD              Maximal tolerated dose

OR                Odds ratio

PI                  Principal Investigator

PK                Pharmacokinetic

PKPD           Pharmacokinetic-pharmacodynamic

RDT              Rapid diagnostic test

SAE              Serious adverse event

SDF              Side stream-dark field

SERU           Scientific and Ethics Review Unit

SMAART     Severe Malaria in African children: A Research and Trials consortium

SOP              Standard Operating Procedures

TAT              Thrombin-AT III

TF                Tissue factor

TM               Thrombomodulin

ULN             Upper limit of normal

## Introduction

Over the last decade there has been an unprecedented rise in funding for malaria control activities, including the scale-up in long-lasting insecticidal bed nets and introduction and access to effective artemisinin-combination treatments (ACT). This has resulted in the disease retreating from large parts of the globe, yet malaria remains stubbornly unyielding in sub-Saharan Africa and in some parts of Asia ^1^. In some African countries, the pattern of transmission has not changed appreciably, despite implementation of early treatment and control strategies and they continue to contribute most to the global disease burden ^2^. Many of the African countries most afflicted by malaria are amongst the poorest on the continent with weak health services infrastructure. Severe malaria remains a major public health problem in Africa and a chief factor in child mortality; particularly in countries experiencing high transmission. In 2016 it was estimated that there were over 445,000 deaths from malaria with the vast majority of these deaths in African children ^3, 4^. Whilst the AQUAMAT trial has provided definitive evidence for optimal antimalarial treatment in severe malaria ^5^, progress on improving outcome through supportive or adjunctive treatments has been very slow ^6, 7^.

### Treatment of severe malaria

The multi-centre AQUAMAT trial conducted in 11 centres in 9 countries in Africa compared quinine and artesunate in 5425 children hospitalised with severe malaria. The primary endpoint, in-hospital mortality, in the intention to treat analysis occurred in 297/2713 (10.9%) children receiving quinine treatment compared to 230/2712 (8.5%) children receiving artesunate - translating to a relative reduction in mortality of 22.5% (95% CI 8.1-36.9) with artesunate (p=0.002) ^5^. However, outside the context of a clinical trial, overall in-patient mortality for severe malaria remains unacceptably high ( ^~^10%), and unlikely to improve without wider implementation of pre-referral artemisinin ^8^ and better supportive treatments ^6, 9^. As demonstrated in Table 1 below, case fatalities in AQUAMAT were even higher within large subgroups of patients presenting with one or more of the 3 key prognostic markers (coma, acidosis, or a high blood urea nitrogen) ^10^. Evidence guiding best management of these and other complications is lacking. To date none of 33 clinical trials of adjunctive (supportive) treatments conducted globally since a seminal severe malaria trial in 1980 have shown benefit ^6^. Over 60% of these trials involved children and 15 were specifically directed to the sub-group with cerebral malaria. The majority were single-centre Phase I or II trials involving few participants, and a reasonable number were stopped prematurely because they showed harm.

**Table 1.** High priority risk factors for severe malaria and recent trials.

Admission feature or complication	Frequency	In-hospital Mortality * (Artesunate-arm) ^5^	
Coma	32–35%	18%	
Metabolic acidosis (base excess<-8 or lactate>5mmol/L)	43–44%	15%	
Renal impairment (Urea/BUN > 20 mmol/L)	24%	22%	
Hypoglycaemia (blood glucose <3 mmol/L)	10%	15% ^11^	
Convulsions	30–32%	14%	
Invasive bacterial co-infection	5.5%	24% (Meta-analysis) ^12^	
Blackwater Fever (region specific)	14–21%	Day-28 mortality 12% **^13^**	
**Recent or ongoing trials**	**Frequency**	**Mortality**	**Trial:** results expected
Shock (mortality = no-bolus arm)	12%	8.5%	**FEAST:** published **^14^**
Severe anaemia	29–30%	1%	TRACT: published ^15, 16^
Hypoxaemia (<90%)	15%–17%	2–13%	**COAST:** published **^17^**

* Data are from AQUAMAT trial unless otherwise stated

### Severe Malaria in African children A Research and Trials Consortium (SMAART)

Severe Malaria In African Children: A Research and Trials Consortium (SMAART) was funded in 2018 by a collaboration award in science from the Wellcome Trust. The major objective of the consortium was to identify which interventions could optimise the whole treatment pathway for children with severe malaria to survival 6-months post-discharge, and hence achieve a step-change in improving their outcomes in the current era. The SMAART consortium reviewed the high priority areas of research and key targets for intervention. One high risk group identified by the group was children presenting with acidosis (increased base excess); in this sub-group mortality remained at 15%, and acidosis was one of the three key factors predicting poor outcome ^10^. In order to identify supportive therapies which could target acidosis, the following section summarises the current understanding of the pathophysiology of severe malaria and likely aetiology of ‘malaria’ acidosis.

### Pathophysiology of severe malaria and metabolic acidosis

During the course of infection, ring stage parasitaemia or infected erythrocytes in children with *P. falciparum* is amplified. Unlike other malarias species, *Plasmodium falciparum* has the unique ability to cause cytoadherence, a phenomenon called sequestration, of late stage parasitised infected erythrocytes in the deep vascular beds ^18^. The pathophysiological process is mediated by excessive sequestration of *P. falciparum* infected erythrocytes ^19^, rosetting ^20^ and decreased deformability of non-parasitised red cells ^21^. Whilst this can occur during a non-severe infection, autopsy studies have shown that there is intense sequestration of parasitized erythrocytes in vital organs in children who have died from malaria (i.e., had severe malaria), but sequestration varies between organs, and even varies even within an organ, with some vessels completely blocked while other proximate vessels remain patent ^21, 22^. Whether sequestration causes mechanical obstruction and impaired tissue perfusion or is damaging in other ways (active parasite metabolism, release of toxins, cytokine induction) ^23^ is not known.

One marker associated with poor outcome is a raised lactate, which is generally considered to be directly linked to the degree of impaired perfusion ^24^. Evidence to support this include the fact that improvements in lactate concentration over the first 24 hours of admission were strongly prognostic for survival in adults with severe malaria ^25^. Moreover, faster clearance of plasma lactate was predictive of the treatment effect on mortality of artemether compared to quinine ^26^. Artemether results in a rapid killing of ring stage parasites, preventing their further maturation and sequestration in the microcirculation and this is thought to be a main contributor to the improvement in case fatality. In the fluid expansion as a supportive therapy (FEAST) trial, which included a large number of children with severe malaria, a sub-analysis showed that severe lactataemia (>5 mmol/L) was strongly associated with mortality (Odds Ratio (OR) 6.96; 95% CI 3.52, 13.76, p<0.001) and that failure to clear lactate at 8 hours was strongly associated with death at 72h (OR 4.62; 95% CI 2.7, 8.0; p < 0.001) ^27^.

### Adjuvant or supporting treatments aiming at improving the microcirculation

The demonstration in autopsy studies of microvascular obstruction by a heavy parasite burden ^28^ and that an overall measure of parasite biomass ( *Plasmodium falciparum* histidine-rich protein2 (pfHRP2), a protein released on sequestration by infected erythrocytes), correlates with worse outcomes ^29^ suggest that adjuvant therapies which can reverse sequestration and reduce overall biomass (by preventing merozoite invasion) early in the course of the disease may lead to substantial improvements when the risk of fatal outcome is highest. Moreover, the time of development of a merozoite into an adhesive infected erythrocyte that sequesters and blocks the micro-vasculature is ^~^18–20 hours which is the same time period (first day of hospitalization) when the majority of paediatric deaths from severe malaria in Africa occur.

In studies conducted in Indonesia, it was hypothesized that heparin could inhibit *P. falciparum* sequestration and merozoite invasion since heparin binds to heparan sulphate binding structure of *Plasmodium falciparum* erythrocyte membrane protein 1 (PfEMP1), the Duffy-binding like domain 1α (DBL1α), known as a vital contributor to sequestration of infected erythrocytes ^30, 31^. Clinical trials showed that heparin, as an adjunctive therapy to antimalarial drugs, had variable results, with some showing reduced mortality in children with severe *P. falciparum* malaria ^32, 33^, others showing no clinical improvements when given as a low dose ^34^ and others demonstrating potentially severe effects on coagulation in simian studies in *P. knowlesei* malaria at the same dosage as those showing benefit in children with severe malaria ^35, 36^. As a result, heparin was not subsequently adopted into clinical practice owing to the substantial concern over haemostatic side effects. Subsequent investigation has shown that that the inhibitory effect of heparin on *P. falciparum* sequestration and merozoite invasion (which also is mediated through the heparan sulphate binding site of PfEMP) is independent of its anti-coagulant activity ^37, 38^. The next step was to develop a heparin compound that was devoid of its therapeutic limiting effects on coagulation.

### SEVUPARIN

The drug sevuparin was developed from heparin because it was necessary to conserve a heparan sulfate binding activity that was as similar as possible to that of heparin. Thus, the rationale was that sevuparin would act as a decoy receptor during malaria infection ^39^. Sevuparin, akin to other heparins, is a poly-disperse chemical, encompassing a range of polysaccharide chain lengths with molecular weights of 3.6–9.6 kDa. Sevuparin is negatively charged and derived from heparin through chemical depolymerization. In sevuparin, the specific pentasaccharide involved in high-affinity binding to antithrombin III has been deleted. Thus, since sevuparin has no specific binding sequence for antithrombin (AT) which is the main contributor to prolonged coagulation, it has no direct effect on factor Xa or on thrombin, and its effect on activated partial thromboplastin time (APTT) is markedly reduced compared to that of standard dose heparin ^40^. For example, for it to have the same effect (ED50) on APTT prolongation (measured as ED50), sevuparin would need to be given in doses five times higher compared to low molecular weight heparin and 35 times higher compared to full length heparin.

Like heparin, sevuparin binds to the heparin sulphate binding structure of Plasmodium falciparum erythrocyte membrane protein 1 (PfEMP1), which is the key receptor for sequestration of infected erythrocytes ^40, 41^. Preclinical investigations have demonstrated that sevuparin blocks *P. falciparum* merozoite invasion into fresh erythrocytes *in vitro*, and both disrupts and blocks the binding of infected erythrocytes to uninfected erythrocytes (rosetting) and binding to vascular endothelial cells (also known as cytoadherence) *in vitro*. *In vivo* studies also demonstrated that sevuparin led to de- sequestering of schizonts both rats and in non-human primate preclinical studies ^32– 34^ and to disruption of rosetting in a dose-dependent manner ^41^.

Phase I studies in healthy volunteers and a clinical study of adults with sevuparin (including a dose escalation trial) have been conducted in patients with mild *P. falciparum* malaria in which it was hypothesised that sevuparin would act as a decoy receptor during malaria infection to block merozoite invasion as an adjunctive therapeutic approach to preclude the early expansion of an infection and reduce the sequestered biomass ^42^.

In the Phase I dose escalation trial, sevuparin was found to be safe and well-tolerated with minimal effects on APTT at doses of 1.5 mg/kg and 3mg/kg every six hours but a dose of 6.0 mg/kg led to higher APTT values. The Data Monitoring Committee (DMC) therefore recommended that the study should continue to the Phase II with a dose of 3.0 mg/kg sevuparin every 6h, given the potentially increased risk of adverse events at higher doses ^42^.

In the Phase II trial, sevuparin was administered as an intravenous infusion over 5 minutes in addition to standard care i.e., a fixed dose combination of 1000 mg atovaquone and 400 mg proguanil once daily for three days, in subjects with uncomplicated *P. falciparum* malaria (experimental arm), as compared to atovaquone/proguanil treatment alone (control). There were minimal and non-clinically relevant changes in anti-Xa- and prothrombin-times, and the international normalized ratios associated to the sevuparin dose. Increases in APTT were dose-dependent and appeared to follow the time-concentration curve for each sevuparin infusion. The 6-hour interval between the infusions allowed for nearly full reversibility of APTT levels after each dose, and no accumulative effects were seen over the course of the 12 consecutive infusions. Thrombocytopenia occurred in one subject but was noted to be present before the initiation of sevuparin treatment. There was no incident of bleeding in any of the participants nor were there any other serious adverse events or adverse events ^42^.

Sevuparin was therefore judged to be safe and well tolerated in adults with mild malaria and was found to safe with minimal side effects in other clinical trials ^43^. It led to a reduction in numbers of ring-stage infected erythrocytes after a single sevuparin infusion and resulted in a transient appearance of mature parasite infected erythrocytes (schizonts appearing in the circulation, indicating these had been released after desequestration). These both occurred within one hour after the first sevuparin injection. Thus, these studies indicate that a novel new drug candidate for adjunctive treatment of severe malaria had been identified that blocks merozoite invasion and transiently de-sequesters infected erythrocytes in humans with uncomplicated *P. falciparum* malaria.

### What is known about coagulation in severe malaria?

Endothelial injury, whether due to trauma, inflammation or infection causes activation of three main pro-coagulant pathways: the coagulation cascade, platelet reactions and vasoconstriction. In severe *P. falciparum* malaria, adhesion molecule upregulation has been demonstrated ^44, 45^ and thrombomodulin levels (TM) have been reported to be high ^45, 46^ suggesting that any coagulation activation seen might be due to endothelial dysfunction. Few detailed studies exist of coagulation abnormalities in severe malaria; frank disseminated intravascular coagulation (DIC) is rare despite thrombocytopenia being common ^47– 49^. Early studies of the mechanisms involved in the activation of the coagulation cascade in severe *falciparum* malaria in Thai adults, many of whom had multiple vital organ dysfunction, suggested activation of the intrinsic pathway of the clotting cascade and complement system including reduction in the concentration of plasma antithrombin III (AT III) concentrations, elevation in thrombin-AT III (TAT) complexes ^50, 51^, and reductions in factor XII and pre-kallikrein activities. Protein-C activity was also shown to be reduced ^51^. Subsequent studies have also shown that *P. falciparum* malaria is associated with procoagulant activity but not with clinical evidence of thromboembolism. Plasma levels of TM have been used to assess the participation of the vascular endothelium in human *falciparum* malaria. Studies in adults have shown that elevated plasma levels of TM correlate directly with the levels of parasitaemia, TNF alpha, elastase and TAT ^46, 52^; and that the low plasma levels of Protein-C and protein-C inhibitor- 1 and increased TAT concentrations present in almost all patients correlated with severity and parasitaemia. Together these data suggest that there is endothelial activation and a shift towards a pro-coagulant state in *P. falciparum* malaria, both of which can be reversed after anti- malarial treatment ^52^.

### Paediatric studies of coagulation in severe malaria

Studies of coagulation in African children with severe malaria are relatively few but clinical evidence of DIC is rare ^53^. In paediatric cerebral malaria (CM), autopsy studies have shown that fibrin degradation products are raised, indicating a pro-coagulant state ^54^. These studies also showed a consistent staining for tissue factor (TF) in the endothelial cells and TF was also shown to be upregulated in the brain post-mortem studies in paediatric CM ^55^. However, the latter study also showed that TF was also found in post-mortem samples from parasitaemic children whose underlying illness was non-malarial ^55^. Moreover, when comparing functional coagulation assays in African children with CM to children with mild malaria, no marked differences were found ^56^.

A more recent and comprehensive case-control study of coagulation compared a range of indices in children with true cerebral malaria (defined by a malarial retinopathy) compared with children with other forms of severe malaria, mild malaria and healthy controls. Compared to healthy controls (n=19), TAT, a sensitive marker of thrombin generation, was increased in children with retinopathy- positive CM (n=66) (P<0.001) and levels were greater than those in children with uncomplicated malaria (n=30) (P<0.01). In the retinopathy-positive CM group, TAT levels were higher in 16 fatalities than in those children who survived. Prothrombin times were mildly and similarly prolonged in both CM children and children with uncomplicated malaria compared to healthy controls ^57^. APTT levels were similar to the controls in all malaria groups, indicating activation of coagulation through TF activation rather than increased factor XII ^51^.

## Protocol

### Trial registration

PACTR number: 202007890194806 (registered on 20/07/2020)

ISRCTN32271864 (was registered on 28/07/2021 and updated on 18/08/2023)

Protocol Version 2.1 Date 28 ^th^ February 2023

This protocol follows the SPIRIT guidelines ^58^.

### Justification for the study

The poor outcomes in children with severe malaria complicated by lactic acidosis indicates that adjunctive therapies directed at the pathophysiology underpinning acidosis may be beneficial. A novel new drug candidate, sevuparin, has been identified that can block merozoite invasion, prevent cytoadherence and transiently de-sequesters infected erythrocytes – the main causes of microvascular blood flow impairment (and likely aetiology of acidosis) If given, in addition to antimalarial treatment, early in the course of admission (<24 hours) this could result in improvements in outcomes from severe malaria for the subgroups at greatest risk and during the period of greatest risk (the first day of hospitalisation). Sevuparin has been shown to be safe and well tolerated in adults with only some mild effects on activated partial thromboplastin time (APTT) at higher doses given over longer periods of time (3 days), which were transient and had no clinical consequences. We propose an initial investigation should be a Phase I trial which is designed to obtain data on safety, dosing, feasibility and potentially lactate clearance of sevuparin as an adjuvant therapy in severe malaria in children.

### Our hypotheses

We hypothesize that sevuparin, a de-polymerised heparan sulphate mimetic, will improve microcirculatory flow by reversing and preventing parasite sequestration when given to children with severe malaria and will improve overall outcome.

## Objectives

### General objectives

The primary objective of this trial is to conduct a dose-finding study of intravenous sevuparin given in 3 doses over the first 18 hours from enrolment (within 24h of hospital admission), defining toxicity events as any APTT >2.5 upper limit of normal (ULN) (grade 3 toxicity) 1 hour after each dose to identify the maximum tolerated dose (MTD). The initial dosage (1.5 mg/kg) and the *a priori* dose- toxicity curve are based upon the results of the adult trial (where a dose of 1.5 mg/kg was associated with minimal risk of toxicity) and experimental evidence of dose-dependent efficacy i.e. inhibition of merozoite invasion and reversal of cytoadherence of infected erythrocytes ^42^. Almost all adults enrolled in this trial experienced grade 2 toxicity after one or more sevuparin doses, but APTT rapidly normalized, hence the choice of grade 3 toxicity to define the MTD in this dose-finding trial.

The primary endpoint for the future Phase II trial reflects the primary hypothesis that Sevuparin improves microcirculatory flow by reversing and preventing parasite sequestration. Data collected in the Phase I trial will also assess whether lactate clearance at 8-, 16- and 24 hours is a realistic and feasible primary endpoint for a subsequent Phase II trial.

### Specific objectives

To identify the maximum tolerated dose (MTD) of intravenous sevuparin as an adjunctive therapy in children with severe malaria given as three infusions at 0, 8 and 16 hours using the Continual Reassessment Method (CRM) to adapt or inform subsequent doses for each child entering the trial, based on a toxicity event defined as any APTT >2.5 upper limit of normal (ULN) 1 hour after each dose, and updating the dose-toxicity model using the previous patients’ APTT results. The secondary objective will be to assess whether lactate clearance at 8 hours is a realistic and feasible primary endpoint for a subsequent Phase II trial.

## Methods

### Study sites

The study will be conducted on the high dependency ward in Kilifi County Hospital, Kilifi Kenya and the paediatric ward at Nchelenge Hospital, Luapula Province, Zambia

### Study design

A Phase I safety and dose finding trial

### Study populations

Twenty children hospitalised with severe malaria

### Inclusion criteria

1.   Aged between 3 months and 12 years admitted to the paediatric wards within the last 24h

2.   Current evidence of *P. falciparum* malaria (slide positive)

3.   Clinical evidence of severe malaria: impaired consciousness: coma (inability to localize painful stimulus) or prostration (inability to sit unsupported for those above 6 months) or deep breathing

4.   Lactate > 2 mmol/L

5.   Guardian or parent willing and able to provide consent

### Exclusion criteria

1.   Clinical evidence or a history of a bleeding/coagulation disorder

2.   A comorbidity which clinician believes has a significant risk of poor outcome e.g., malignancy, end-stage renal failure, major cardiac condition

3.   Thrombocytopenia (platelet count <25 ×10 ^9^/L).

## Sampling

### Sample size determination

This is a Phase I trial designed to obtain data on safety, dosing, feasibility, and lactate clearance of sevuparin given as an adjuvant therapy in severe malaria. We aim to study 20 children since this will allow sufficient data on safety to be generated across a range of doses to identify the maximum tolerated dose (MTD) from a more informed model relating dose to toxicity events (denoted the ‘dose- toxicity’ curve) than that available *a priori* based on published data from adult studies. After each patient is enrolled, the dose-toxicity curve will be updated based on levels of APTT taken over three time points (1h post each infusion), defining a toxicity event as APTT >2.5xULN at any time point (grade 3 following the Common Toxicity Criteria (CTC)). This enables the MTD to be estimated more rapidly using the Continuous Reassessment Method (CRM) ^59^ once determined, subsequent participants will be allocated to this MTD to provide the most accurate estimate of future toxicity event rates until we reach the sample size of 20 children. However, the CRM method will continue to use information from all these children; for example, if a number of children receiving the originally identified MTD experience toxicity events, the dose would again be lowered, and future children would receive this lower dose.

***Sampling procedures and methods.*** Children admitted to the paediatric wards of in Kilifi and Nchelenge Hospital children with suspected malaria and either coma or deep breathing (defining severe malaria for this Phase I study) will be screened by the paediatric triage/admission team for eligibility. Once eligibility is verified, the parents can be approached for consent and, if they agree to participate, they will receive their allocated treatment dosage. The blood tests taken at admission and during the trial include standard of care and research bloods (see Table 2). Admission and serial assessment of full blood count, admission point of care clinical chemistry including PH, blood cultures and repeated assessment of malaria parasitaemia are part of the standard clinical tests. Additional to this will be serial assessment of lactate, measurements of coagulation (by iSTAT (Kaolin ACT) and laboratory-based APTT) and samples will be stored in Kilifi for future pharmacokinetic (PK) tests, plasma HRP2 tests and malaria parasites(research). The reliability of Point of care ACT measurements (using the iSTAT) have previously assessed against a laboratory gold standard and have shown that the coefficients of variation of POC ACT and whole blood were between 2% and 3.6%, indicating that POC assessments are reliable and able to support on-site decision-making for patients in acute and intensive care ^60^.

Management and outcome data will be collected (clinical parameters and recovery, developmental assessment, number of transfusions, use of drugs (specifically anticonvulsants, paracetamol, and antibiotics), date of discharge or in-hospital death. Contact and locator data will be recorded so that children can be followed at day 7 and day 28.

***Consent process.*** Once eligibility has been confirmed, authorized trial staff will approach parents/guardians to invite their child to take part in the trial. An information sheet will be provided to the parent/guardian in their usual language. The sheet will be read aloud to those who are unable to read. The doctor/nurse will check that the information has been fully understood and parents/guardians will be encouraged to ask questions they may have about their child’s participation.

Where possible, prospective written informed consent will be sought from parents/guardians by asking them to sign the Consent Form. If parents/guardians are unable to sign, a thumbprint will be taken in lieu of a signature. A copy of the Consent Form will be given to the parent/guardian, the original placed in the patient’s medical notes, and a copy kept in the Investigator Site File.

If it is considered that the full consent process would significantly delay enrolment, and consequently be detrimental to the child’s health, then emergency verbal assent, used in previous trials ^61^, will be sought from parents/guardians by the admitting medical team. Following verbal assent, written informed consent will be sought from the parent as soon as possible once the child’s clinical condition has stabilized.

The participant information sheet, consent form and case report files can be found as *Extended data* ^62^.

**Figure 1.**
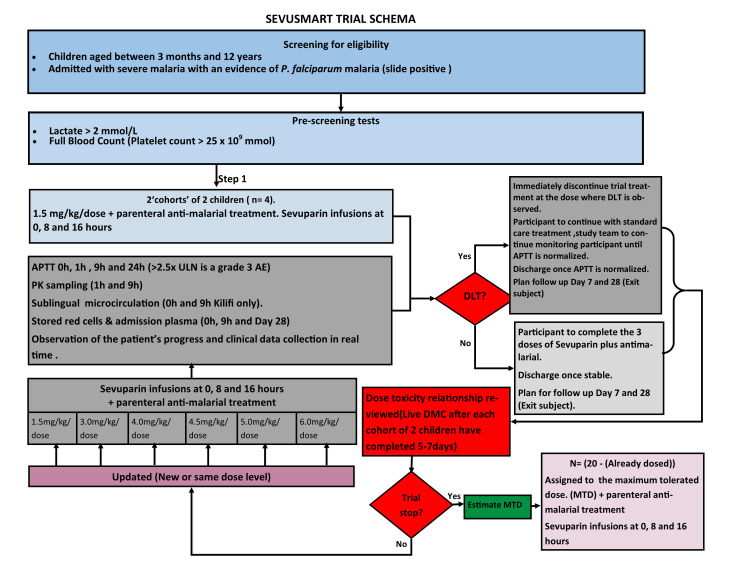
Trial Flow

***Trial treatments.*** We aim to study 20 children (See Figure 1 Trial Flow). The trial will be open label with clinicians aware of study drug dosage. Laboratory staff performing APPT levels will be blind to drug dosage. All children will receive standard care including parenteral artesunate. Sevuparin will be given as three infusions at 0, 8 and 16 hours after enrolment. The initial participants (two ‘cohorts’ of 2 children each, i.e., 4 children in total) will receive a dose of 1.5 mg/kg/dose with the plan to escalate up to the next highest dose up to a maximum of 6.0 mg/kg/dose for subsequent children. In order to determine whether, and the rate of escalation, to move to a higher dosage for subsequent patients after the first 4 children we will use a design called the Continuous Reassessment Method (CRM). This starts with an *a priori* dose-toxicity curve, reflecting the probability of experiencing a toxicity event (here APTT>2.5xULN 1h after any of the three doses) to the dose received. In this method of dose finding, the dose-toxicity curve is re-fitted to the data after each child is enrolled, based on their observed dose and whether they had a dose limiting toxicity event. After the first 4 children, each subsequent patient would be assigned the next highest dose, until the estimated risk of toxicity is just below or at the target toxicity level, designated as the maximum tolerated dose (MTD). The dose limiting toxicity (grade 3 APTT, >2.5xULN), *a priori* dose-toxicity curve and chosen target toxicity rate (15%) used in this trial have been based upon data shared by the investigators from the adult sevuparin studies in Thailand [36]. In particular, almost all adults enrolled in this trial experienced grade 2 toxicity after one or more sevuparin doses, but APTT rapidly normalized, hence the choice of grade 3 toxicity to define the MTD in this dose-finding trial. APTT 24h post-enrolment is a key secondary safety endpoint to confirm rapid normalization.

Any child with APTT>2.5xULN 1h after a dose of sevuparin will immediately discontinue trial treatment (and it would be counted as a toxicity event). They will continue to be followed according to the trial schedule (on-study, off-study-drug), to confirm resolution of APTT and to record clinical outcomes.

### Clinical assessments

Members of the clinical team and study team will all receive pre- and peri-trial training on the management of severe malaria. A manual of operations for the trial will be available for the study team, to anticipate and troubleshoot any potential issues. Vital signs will be monitoring regularly (including temperature, heart rate, respiratory rate, blood pressure, oxygen saturation, conscious level). These will continue twice-daily after 24 hours until the child is discharged from HDU. All children will be managed on the HDU until conscious and able to take and retain oral fluids/food. During the period of sevuparin administration (0–24 hours) children in Kilifi, Kenya will have continuous electrocardiogram (ECG) monitoring for safety. However, it is notable that the trial in Thai adults showed no signs of QT prolongation ^42^. Once discharged from HDU children will be reviewed daily until discharge and followed up at day 7 and day 28. On admission and Day 28 children will be assessed by an adapted Kilifi Developmental Index to assess developmental status and clinically for neurodevelopmental sequelae (see Table 2). Non-compliance is limited by the intervention being administered by clinical teams during admission. Any child who develops APTT>2.5xULN (grade 3 toxicity) will not receive further doses of sevuparin but will continue to be followed up. Children lost to follow-up before day 28 will be traced for vital status (permission requested within consent) using locator data and multiple contact phone numbers recorded before discharge.

**Table 2.** Clinical management schedule.

	Assessment Time										
Procedure	Adm	1h	2h	4h	8h	9h	17h	24h	Bi daily to discharge	Day 7	Day 28
Clinical assessment	X	X	X	X	X			X	X	X	X
ECG continuous to 24 hours (Kilifi)	X							X			
APTT and coagulation tests (ACT) (1.7ml)	X	X 1 hr post dose				X 1 hr post dose	X 1 hr post dose	X			
Microperfusion ^&^	x					x	x				
Lactate (point of care)	X	X				X		X			
Standard clinical test non-research (FBC, POC chemisty Istat) *		X							X		X
Malaria slides	X					X		X	(36,48, 72 hrs)	X	X
Stored red cells & admission plasma **	X					X					X
PK sample		X				X					
Neuro-developmental assessment	X										X

^&^ Kilifi Site only

* Standard- non research clinical tests full blood count, clinical chemistry POC iSTAT (electrolytes, pH BUN) will be done at admission and at 24 hours and Day 7 (Full blood count only).

Other laboratory test: venous blood gases (including base excess) will be done at 0, 9 and 24 hours

Malaria slide and morphology will be done at 0, 9, 17, 24, 36, 48 hours (and 72 hours if in hospital); at follow up (Day 7 and 28)

**For quantitative plasma HRP2 assessment, biomarkers and parasite morphology (Kilifi site only)

### General clinical management

Children will initially receive parenteral antimalarial treatment (artesunate), followed by on day 3 (or when the child can safely take and retain oral feeds and fluids) an oral course of artemisinin combination therapy (ACT). All trial patients will receive intravenous antibiotics. Intravenous maintenance fluids will be given at a rate of 4ml/kg/hour until the child is able to drink and retain oral fluids. Antipyretics, anticonvulsants and treatment for hypoglycaemia and other treatments will be given as clinically required and will be administered according to nationally agreed protocols. Children with Hb <4 g/dl (or Hb <6 g/dl and respiratory distress) will be transfused with 20mls/kg of whole blood as soon as blood is available. In the absence of blood, standard care as per local treatment guidelines will be followed.

### Protocol treatment discontinuation

An individual patient may stop treatment early or be stopped early for any of the following reasons:

▪ Unacceptable toxicity or adverse event
▪ Intercurrent illness that prevents further treatment
▪ Any change in the patient’s condition that justifies the discontinuation of treatment in the clinician’s opinion
▪ Withdrawal of consent for treatment by the patient or parent.

Participation in the trial is entirely voluntary, and parents, carers or older children may choose to discontinue the trial treatment at any time without penalty or loss of benefits to which they are otherwise entitled. Although not required to give a reason for discontinuing their trial treatment, a reasonable effort should be made to establish this reason while fully respecting the patient's rights.

Patients should remain in the trial for the purpose of follow-up wherever possible (unless the patient withdraws their consent for follow-up). If a patient withdraws from the trial, the medical data collected during their previous consented participation in the trial will be kept and used in analysis. This will also apply to parents/carers who withdraw from the trial after assent, that have not completed the deferred consent process. Consent for future use of stored samples already collected can be refused when leaving the trial early (but this should be discouraged and should follow a discussion). If consent for future use of stored samples already collected is refused, then all such samples will be destroyed.

### Trial products, storage and accountability

The drug product, sevuparin 150 mg/mL solution for intravenous (IV) infusion, is formulated in a 0.015M phosphate buffer at a pH of 7.0. It requires storage in a refrigerator at 2–8° and to be protected from light. The non-preserved sterile solution needs to be dispensed (5.4 mL) in a glass vial sealed with a rubber stopper and covered with a tear-off aluminum cap. The solution for administration will be prepared in a syringe and will be kept refrigerated and used within 24 hours. One vial will only be used for one subject. The drug product is produced in compliance with current Good Manufacturing Practice (GMP). The same material and compositions has been used in a clinical trial in children with sickle cell disease ^43^. from the product has been donated by MODUS Therapeutics, Sweden for use in this trial.

## Sub studies

### Assessments of the pharmacokinetics of Sevuparin

An aliquot of plasma from the samples taken during the trial will allow pharmacokinetic- pharmacodynamic (PKPD) modelling of the relationships between drug levels and longitudinal APTT and plasma lactate levels. The evaluation of the PK data will focus on the association between the sparsely sampled sevuparin concentrations and the APTT levels, plasma lactate levels and renal function. These assays will be contracted to Accelera (Nerviano (MI), Italy) since they have validated methodology for Sevuparin measurement and the data sent to MORU, Bangkok who will undertake tbe PK modelling at the end of the clinical study.

### Assessment of microcirculation

The sublingual microcirculation will be assessed at the Kilifi site only using incident-dark field (IDF) imaging (CytoCam, Braedius Medical BV ^®^). IDF orthogonal polarization spectroscopy imaging technology is a validated real-time visualization of the microcirculation using a 2ms pulsed green light emitted at a 530nm wavelength for optimal optical absorption by the haemoglobin in red blood cells, independent of oxygenation state. It is safe, non- invasive and can be performed rapidly at the patients’ bedside. A sterilised disposable lens is used to prevent contact between the instrument and patient and therefore to prevent any transmission of infection. Two trained individuals will collect this data to limit inter-user variability. Images will be recorded from the proximal, mid, and distal portions in each halfof the sublingual mucosa and averaged. Where possible the microcirculation will be assessed at time 0 (prior to sevuparin dose), at 8–9 hours (before and after infusion) and again at 17–18 hours (after final dose). The image acquisition and analysis used for assessment of the microcirculation will be in line with the 2018 consensus agreement. This will include a Capillary Network Analysis for vessel densities (total and perfused), the proportion of perfused vessel and the average perfusion speed indicator.

### Additional laboratory investigation

At 1- and 8- hours and Day 28 plasma and red cell pellets will be used measure of the level Plasmodium falciparum Histidine Rich Protein (plasma). In addition the presence of rosetting and detailed microscopic examination of the infected red-cell morphology to stage the maturity of the parasite (Kilifi site only).

## Trial outcome measures

### Primary outcomes

APTT>2.5xULN 1h post any sevuparin dose (grade 3 following the CTC)

### Secondary outcomes

Change in lactate from 0 to 8 hours

Presence of mature infected erythrocytes on the blood films at 8 and 24 hours

Parasite clearance time

(Change in sublingual microcirculation over time)

### Safety endpoints

APTT 24h post enrolment (absolute level and grade)

Development of abnormalities of coagulation indices (prothrombin) (Grade 2 and above)

Neurological sequelae through day 28

Mortality through day 28

Serious adverse events through day 28

Grade 3/4 adverse events through day 28

APTT 24h post enrolment will be used as an assessment of normalization 8h after the final sevuparin dose. Both absolute levels and grade will be considered.

De-novo evidence of neurological sequelae will be ascertained using a modified Kilifi Developmental Index ^63^ assessed at admission (to identify pre-existing conditions) and follow up (which we have adapted to use for the other trials.

Serious adverse events will use the standardized definitions (see section 11 below). SAEs will be independently reviewed in real-time by the DMC. Adverse events will be graded following the Common Toxicity Criteria v5.0: ( https://ctep.cancer.gov/protocoldevelopment/electronic_applications/docs/CTCAE_v5_Quick_Refe rence_5x7.pdf).

### SAEs and interim analyses

SAEs will be reviewed immediately by a designated physician (SAE reviewer) and reported to the appropriate ethics and regulatory committees within one week. The Chief Investigator will inform the Trial Steering Committee (TSC) and Data Monitoring Committee (DMC) for review on a regular basis (as deemed necessary). The DMC will review data after the first 2 ‘cohorts’ of 2 children each have been enrolled to the lowest dose, and then after each Dose Limiting Toxicity event. Professor Timothy Peto (Oxford) has agreed to be the Chairman is the DMC, other members will include an independent statistician and 2 pediatricians with relevant DMC or clinical trial experience. They will meet by regular teleconference. The continual reassessment method uses data from each enrolled participant to update the dose- toxicity curve and then suggests an escalation of dose to the largest dose with risk of estimated risk of toxicity below the target toxicity level if appropriate.

***Trial monitoring.*** The trial will be monitored by the Clinical Trial Facility in Kilifi oversees standards and quality of all trials conducted through the KWTP and through its monitoring systems and standard operating procedures are organised to ensure that all sites can be monitored with equal independence and rigor. All monitors will be appropriately qualified and trained. At each monitoring visit the monitors will:

▪ verify completeness of Trial Master File
▪ confirm adherence to protocol
▪ review eligibility verification and consent procedures
▪ look for missed clinical event reporting
▪ verify completeness, consistency and accuracy of data being entered on CRFs
▪ evaluate drug accountability
▪ provide additional training as needed

The monitors will require access to all patient medical records including, but not limited to, laboratory test results and prescriptions. The investigator (or delegated deputy) should work with the monitor to ensure that any problems detected are resolved.

### Data management

All clinical and laboratory data will be recorded in the CRF and stored with a unique serial number identifier. Data will be entered onto Open Clinica. All data will be regularly backed up and backup copies stored both on and off site. Paper records will be archived in locked cabinets. These cabinets will have limited access with prior authorisation. All data will be partially- anonymized prior to presentation or publication of any results. Archive documents will be sent for long term storage (10 years) at an appropriate facility according to national policies.

### Confidentiality

Participant’s identification data will be required for the registration process. All clinical and laboratory data will be recorded in the CRF and stored with a unique serial number identifier. Information will only be made available to those caring for the child and those directly involved with the study. Data will be entered onto Open Clinica (FDA approved, web-based application). All data will be regularly backed up and backup copies stored both on and off site. Paper records will be archived in locked cabinets. These cabinets will have limited access with prior authorisation. All data will be partially- anonymized prior to presentation or publication of any results. All clinical data will be held confidentially, and personal identifiers will be removed before analysis of the data and presentation of the results.

### Data sharing

After completion of the study, requests for data access from researchers outside the study team will be considered by a subgroup of the Centre Scientific Committee (Data Governance Committee), and where indicated, requestors will be asked to develop scientific protocols for approval of secondary analyses. The potential to share data will be included in the participant Information and Consent Form.

### Statistical analysis

Clinical data will be summarized using means and medians where appropriate for continuous data depending on the distribution. Primary and secondary endpoints will be described using means or medians or proportions. Analyses will follow intention-to-treat. As this is a Phase I trial no subgroup analyses are planned.

### Ethics statement

Ethical approval has been obtained from Kenya Scientific and Ethics Review Unit (SERU), Nairobi Kenya on 25th February 2019 (protocol number GCMR-C/127/3744), Imperial College Research Ethics Committee (ICREC) on 13 ^th^ July 2021 (protocol number 18IC4513) and National Health Research Ethics Board, Lusaka, Zambia on 24 ^th^ July 2023 (protocol number NHREB001/24/07/2023). The trial was registered on the Pan Africa Clinical Trials PACTR number: 202007890194806 on 20/07/2020) and ISRCTN reference no 32271864 on 28/07/2021. These were updated on 18 ^th^ August 2023.

### Safety

The study will be performed in patients who may potentially benefit from the treatment. The risks of cannula insertion and blood drawing include pain, infection at the site of the cannula and thrombophlebitis. These will be minimised by careful technique according to a standard SOP, cannula site inspection and replacement or removal where necessary. No more than 1ml/kg of blood will be drawn for research at any one time. The trial will be recruiting patients with severe illness and likely a high mortality rate. At the start of the trial, the site will receive appropriate training on the use of Sevuparin and will have 2 dedicated clinicians. Sevuparin can lead to minimal and non- clinically relevant changes in APPT (grade II toxicity) In the Phase I dose escalation trial, sevuparin was found to be safe and well-tolerated with minimal effects on APTT at doses of 1.5 mg/kg and 3mg/kg every six hours but a dose of 6.0 mg/kg led to higher APTT values ^42^. The Data Monitoring Committee (DMC) therefore recommended that the study should continue to the Phase II with a dose of 3.0 mg/kg of sevuparin every 6h, given the potentially increased risk of adverse events at higher doses.

Risk will be minimised by the trial design/methods as described above under trial treatments. The escalation through the doses to find the maximum tolerated dose using the dose toxicity curve and data from children previously enrolled is carefully monitored by the DMC and Investigators.

### Benefits

All patients will be closely monitored so that clinical deteriorations can be identified at the earliest opportunity and appropriate therapy initiated. Prior to the start, the dedicated study teams will undergo detailed training on general management of severe malaria and its complications and receive very detailed training on the use of sevuparin. We believe this will afford all children enrolled in the trial with a higher quality of care. All routine non-trial medications required by the hospital to treat the child will be made available. Hospital bills for participants will be covered by the study (covering the costs for standard treatment for severe malaria and related complications). The parents or guardians for the children will be asked to return for a follow up clinic visit at day 7 and day 28 and thus will be offered continuing care for intercurrent illness, including any investigations or blood tests that are clinically indicated.

## Plans for dissemination of the study outcomes

### Public engagement

Community engagement will be through regular meetings with the community involving community representatives and county Health teams. At these meetings, information and feedback will be given and received. Information arising from the study will be fed back through hospital-wide meetings. This is a Phase I trial of an emergency intervention where our engagement has been at a scientific rather than public/community level. If a larger platform trial arose from this study, we aim to develop a dedicated and informed engagement strategy as part of this future trial. In general, we plan to feed into existing community engagement mechanisms. We aim to build general community awareness of research processes at the local hospital, and support community representative inputs into decisions around research design, consent procedures, patient information and trial conduct.

### National and international policymakers

When the results are available, we will provide a summary briefing highlighting the trial results and what then next steps will be. The current study will go some way towards addressing whether sevuparin is safe in severe malaria and inform the optimal dose to be investigated in a future trial. The results will be published in an open access journal.

## Discussion

A novel drug candidate for adjunctive treatment of severe malaria, sevuparin, has been identified. This has been shown to block merozoite invasion, prevent cytoadherence and transiently de-sequester infected erythrocytes in adults with uncomplicated *P. falciparum* malaria. If given, in addition to antimalarial treatment, early in the course of admission (<24 hours) to children this could result in improvements in the outcome from severe malaria for the subgroups at greatest risk and during the period of greatest risk (first day of hospitalisation). Sevuparin has been shown to be safe and well tolerated with only some mild effects on APTT levels at higher doses given over a longer period of time (3 days), which is not clinically relevant to the time period of greatest risk (first day of hospitalization) ^42^. In this Phase I trial dose-finding paediatric study, we aim to use 3 doses given at: admission (0 hours), and then 8 and 16 hours subsequently, and we will probable dose-limiting toxicity, APTT, 1 hour after each dose (to assess maximum toxicity). The normal ranges of APTT in children have been shown to be the same those in as adults ^64^ so this study will learn from and build upon what has already been published on sevuparin in adults with malaria. A large comprehensive study of coagulation abnormalities in African children with severe malaria, mild malaria and healthy controls demonstrated that there are no derangements in APTT in children with severe malaria compared to mild malaria and healthy controls ^57^, providing reassurance that the comparison of APTT levels with normal ranges in this study is clinically meaningful in terms of identifying a maximal tolerated dose (MTD).

The design of this dose finding study uses the Continual Reassessment Method (CRM). This adaptive dose-finding study design is increasingly embraced by clinical trialists ^65^ as a more efficient method for identifying an “optimal” dose using as small a number of participants as possible, in contrast with heuristic methods such as, for example, comparing three arbitrarily chosen doses. The CRM ‘learns’ (i.e., reassesses risk/toxicity) after each patient is entered into the trial and proposes a subsequent dose for the next child entered in a way that provides the most information about doses closest to the MTD. CRMhas been shown to incur fewer toxicity events overall in identifying the MTD, and to estimate the MTD more accurately as compared the standard Phase I dose escalation designs ^59^. In terms of safety, the study will be run using a ‘live’ Data Monitoring Committee (DMC) review of toxicity events. The DMC will review data after the first 2 ‘cohorts’ of 2 children each have been enrolled to the lowest dose (1.5 mg/kg/dose), and then after each Dose Limiting Toxicity (DLT) event.

The results of this Phase I trial will identify the final dosage selected for a subsequent Phase II that will include both efficacy and safety outcomes. The Phase II trial will be conducted by the SMAART consortium and plans to use change in lactate at 8 hours as its primary endpoint; likely secondary endpoints include neurological sequelae, day-28 and day-180 mortality, length of initial hospitalisation, re-admission to hospital, grade 3/4 and serious adverse events. This Phase I trial will assess whether change in lactate at 8 hours is a realistic and feasible primary endpoint for a subsequent Phase II trial. These data will inform the need for a future Phase III trial with mortality as the primary endpoint.

## Trial Status

The trial opened for enrollment in Kenya on 21 ^st^ Mar 2022. Batch expiry of sevuparin resulted in the following halts to the study: between 01 ^st^ May 2022 and 19 ^th^ May 2022; and 01 ^st^ Nov 2022 to 09 ^th^ Dec 2022 halt. Trial enrolment in Kenya was halted on 30 ^th^ Jan 2023, following the proposed amendment. Enrollment is expected to start in Nchelenge in October 2023.

## Protocol version changes

Version 1.0 was the original protocol submitted for ethical approval to Imperial College Ethics Committee (ICREC) version 1.2 was given full approval on 13 ^th^ July 2021. The original protocol submitted to SERU was version 1.0 dated 18th May 2018, following comments from their reviewers the revisions were included and Version 1.2 dated 8th Feb 2019 was approved. Upon submission to the regulatory body (Pharmacy and Poisons Board), a couple of recommendations resulted in version 1.3 dated 2nd Sep 2020 was approved. ICREC granted a full approval of Version 1.3 on 17th Aug 2021. Version 2.0 and 2.1 included an additional site in Zambia and some changes to the blood collected to assay APPT. Version 2.0 and 2.1 received ICREC 's approval on 27th Mar 2023.

## Data Availability

No underlying data are associated with this article. Imperial College Research Data Repository: SEVUSMART extended data. https://doi.org/10.14469/hpc/13274 ^62^. This project contains the following extended data: ICF SEVUSMART_v1.2 dated 28th Feb_2023_Clean English Copy.doc SEVUSMART Merged CRF's 16th May 2023.pdf Imperial College Research Data Repository: SPIRIT checklist for ‘SEVUparin as a potential Adjunctive Treatment in children with severe malaria : A Phase I trial Safety and Dose Finding Trial (SEVUSMAART)’. https://doi.org/10.14469/hpc/13275 ^58^. Data are available under the terms of the Creative Commons Zero "No rights reserved" data waiver (CC0 1.0 Public domain dedication).
