# Supplementary material for: SEVUparin as a potential Adjunctive Treatment in children with severe malaria: A phase I trial safety and dose finding trial (SEVUSMAART)
Source: Wellcome Open Res. 2024 Aug 12;8:484. Originally published 2023 Oct 20. [Version 2] doi: 10.12688/wellcomeopenres.20111.2 (PMC11362743; doi:10.12688/wellcomeopenres.20111.2)
Supplement: Peer review history

# Reviewer report: Reviewer response for version 2

**DOI:** 10.21956/wellcomeopenres.25245.r93306

**Published:** 2024-08-29

**Authors:** Bruno Mmbando

**Affiliations:** 1 Muhimbili University of Health and Allied Sciences, Dar es Salaam, Tanzania; 2 Public Health, Kampala International University - Dar es Salaam College, Dar es Salaam, Dar es Salaam, Tanzania; 3 Tanga Research Centre, National Institute for Medical Research, Tanga, Tanzania

I have no further comments to make.

Is the study design appropriate for the research question?

Yes

Is the rationale for, and objectives of, the study clearly described?

Yes

Are sufficient details of the methods provided to allow replication by others?

No

Are the datasets clearly presented in a useable and accessible format?

Yes

Reviewer Expertise:

Epidemiology and Statistics

I confirm that I have read this submission and believe that I have an appropriate level of expertise to confirm that it is of an acceptable scientific standard.

# Reviewer report: Reviewer response for version 1

**DOI:** 10.21956/wellcomeopenres.22271.r74237

**Published:** 2024-08-29

**Authors:** Joerg J. Moehrle

**Affiliations:** 1 Medicines for Malaria Venture, Geneva, Geneva, Switzerland

Study proposal is a phase Ib study to assess the safety of sevuparin in children with severe malaria. The primary safety endpoint is the incidence of  APTT > 2.5 LN 1h post infusion of sevuparin.  

 Secondary endpoints are change of lactate levels, presence of mature infected erythrocytes in peripheral blood, parasite clearance time and chage in sublingual microcirculation.

 It is a well-designed protocol to address the still high burden and mortality in children with severe malaria, even if these children receive the best available anti-parasitic drug,  injectable artesunate.

**My comments are:**

1.  Sevuparin is not a registered drug and I couldn’t find any information on the availability of pre-clinical and clinical safety data, especially in children. The protocol also does not provide any information on the availability of preclinical data in juvenile animals. As the authors propose to proceed into children with severe malaria, a  very vulnerable patient group, it would be important to ascertain, that the IMP has been tested in the relevant juvenile tox studies and found to be of no concern.
2. I have not found any assessment of potential interactions between sevuparin and artesunate (either on PK of both drugs  and the antiparasitic effect of artesunate and DHA). It would be important for the review of the protocol, if either simulations,  pre-clinical drug-drug interaction (DDI) studies or clinical DDI studies in healthy volunteers were available to exclude any risk for DDI, especially for a reduced exposure of artesunate and DHA when administered with sevuparin.
3. This protocol is based on  previous studies in adults where “mild malaria” was treated with atovaquone/proguanil and sevuparin; and no negative effects were reported. I have several questions to this approach and its translation to support the proposed study.
  a) What was the definition of mild malaria, what was the number of parasites/ul. Patient symptoms
  b) Atovaquone/proguanil has quite different pharmacodynamic characteristics to injectable artesunate as this combination acts very slowly and even the onset of parasite elimination  and symptom relief. has a lag of 24-48h.

For these reasons, I think that the rationale to move at this stage into vulnerable children is weak. The investigators should consider a pilot cohort where the safety, antimalarial effects and drug drug interactions  are studied in patients with uncomplicated, but hyperparasitemic malaria and OR in an adaptive design study starting with patients >12 years and moderate severity, similar to the study “To Evaluate Efficacy, Safety, Tolerability and PK of Intravenous Cipargamin in Participants With Severe Plasmodium Falciparum Malaria” (ClinicalTrials.gov NCT04675931). If the outcome of this pilot cohort is confirming the tolerability, the beneficial effect of sevuparin on cytoadherence and the lack of significant drug drug interactions (PK as well as PD interactions), then inclusion of children with severe malaria and acidosis is justified.

 I agree with proposed the two step process for obtaining consent for the study participation, however I would recommend that the abbreviated, oral information and consent should also be documented by signing (of fingerprint). Such an approach previously used in severe malaria trials (Kremsner et al. J Infect Dis. 2012 Jan 15;205(2):312-9). Whenever possible assent of the pediatric patient should also be documented.

**Minor comments:**

 The focus of the introduction is on the global burden of malaria and less on the burden of severe malaria and the high medical need to improve the standard of care to reduce mortality in children receiving adequate antimalarial treatment, ie injectable artesunate and therefore should be updated.

 Although the mortality has not changed significantly from the numbers provided in the introduction based on the WHO Malaria Report 2016,   the current numbers published by the WHO should be cited and referenced in the World Malaria Report 2023 ( https://iris.who.int/bitstream/handle/10665/374472/9789240086173-eng.pdf?sequence=1)

**It might also be informative to add to the introduction:**

- What is the percentage of P falciparum infected children that develop severe malaria and especially acidosis
- Impact on K13 mutations on inj artesunate efficacy and increased risk of complications, even if parasite elimination half life remains at 6.5-7.5h, more sequestration?

Is the study design appropriate for the research question?

Partly

Is the rationale for, and objectives of, the study clearly described?

Partly

Are sufficient details of the methods provided to allow replication by others?

Partly

Are the datasets clearly presented in a useable and accessible format?

Yes

Reviewer Expertise:

malaria; drug development; translational research, PKPD modelling, epidemiology, pharmacology, infection biology; clinical trials;

I confirm that I have read this submission and believe that I have an appropriate level of expertise to confirm that it is of an acceptable scientific standard, however I have significant reservations, as outlined above.

# Reviewer report: Reviewer response for version 1

**DOI:** 10.21956/wellcomeopenres.22271.r74240

**Published:** 2024-04-05

**Authors:** Bruno Mmbando

**Affiliations:** 1 Muhimbili University of Health and Allied Sciences, Dar es Salaam, Tanzania; 2 Public Health, Kampala International University - Dar es Salaam College, Dar es Salaam, Dar es Salaam, Tanzania; 3 Tanga Research Centre, National Institute for Medical Research, Tanga, Tanzania

This is phase I clinical trial aiming to determine the safety, feasibility and maximum tolerated dose of intravenous sevuparin as adjuvant therapy in children with severe malaria in children aged 3month to 12 years in Kenya and Zambia. 

 This is well written protocol with detailed literature and methodology to implement the trial. However, the presentation could be improved if more details could be provided as follows:

 1. Out of 20 participants how many will be recruited from Zambia/Kenya? Is this competitive?

 2. Table 2: FBC will be done at 1hr, although in the Figure 1, this will be used for screening. 

 3. It is not how the cohorts of the two participants will be enrolled (parallel of sequentially?

 4. Figure 1: The trial flow is not straight forward to readers. e.g. in the box 3 - step 1 (2'cohorts of 2 children (n=4), this indicates that sevuparin infusion will be given for hours 0, 8 and 16 before decision whether it is safe or not. I would think this should only include hour 0, while the rest (hours 8 and 16) should appear in box 5 (a box with different dosages).  Box 5 should not include hour 0.

 5. Entries for the other cohorts besides the first 2 is not shown in the flow chart. 

 Other minor

 1. Trial monitoring: This should be revised. Suggest to include a word "which" between Kilifi and oversees. Delete the word "are" between procedures and organised.

 2. There is duplicate information between Data Management and Confidentiality. Please rearrange appropriately. See this semi-paragraph "All data will be regularly backed up and backup copies stored both on and off site. Paper records will be archived in locked cabinets. These cabinets will have limited access with prior authorisation. All data will be partially- anonymized prior to presentation or publication of any results"

 3. Some abbreviations are missing, eg FBC and POC

 4. Clinical assessment. Line no5 suggest to change word "monitoring" to "monitored"

Is the study design appropriate for the research question?

Yes

Is the rationale for, and objectives of, the study clearly described?

Yes

Are sufficient details of the methods provided to allow replication by others?

No

Are the datasets clearly presented in a useable and accessible format?

Yes

Reviewer Expertise:

Epidemiology and Statistics

I confirm that I have read this submission and believe that I have an appropriate level of expertise to confirm that it is of an acceptable scientific standard, however I have significant reservations, as outlined above.

# Response

**Published:** 2024-08-02

**Authors:** Kathryn Maitland

**Affiliations:** KEMRI-Wellcome Trust Research Programme, Kenya

We thank the review for a detailed appraisal of our trial protocol and particularly for identifying areas that needed correction. Here are our point by point responses to reviewer:

 1. Out of 20 participants how many will be recruited from Zambia/Kenya? Is this competitive? **Response: **The recruitment at each site is not prescribed and when 20 are enrolled overall we will stop enrolment.

 2. Table 2: FBC will be done at 1hr, although in the Figure 1, this will be used for screening. **Response: **Thank you. The table has been amended. FBC at admission.

 3. It is not how the cohorts of the two participants will be enrolled (parallel of sequentially?)

**Response: **Enrolled sequentially

 4. Figure 1: The trial flow is not straight forward to readers. e.g. in the box 3 - step 1 (2'cohorts of 2 children (n=4), this indicates that sevuparin infusion will be given for hours 0, 8 and 16 before decision whether it is safe or not. I would think this should only include hour 0, while the rest (hours 8 and 16) should appear in box 5 (a box with different dosages).  Box 5 should not include hour 0.

**Response: **The Trial flow demonstrates how the patient will be management. The top section details about whether there is or is not a Dose limiting toxicities will stop result in the patient not receiving additional doses The lower half details the Data Monitoring Committee decisions after reviewing the data and whether to recommend it is safe to escalate to the next dosage.

 5. Entries for the other cohorts besides the first 2 is not shown in the flow chart. 

**Response: **Please see the explanation given for the above. We aim to study initially 1.5 mg/kg/dose with the plan to escalate up to the next highest dose up to a maximum of 6.0 mg/kg/dose for subsequent children. In order to determine whether, and the rate of escalation, to move to a higher dosage for subsequent patients after the first 4 children we will use a design called the Continuous Reassessment Method (the flow chart should be read alongside the details given under Trial treatments section).

 Other minor

 1. Trial monitoring: This should be revised. Suggest to include a word "which" between Kilifi and oversees. Delete the word "are" between procedures and organised.

**Response: **Thank you we have corrected this.

 2. There is duplicate information between Data Management and Confidentiality. Please rearrange appropriately. See this semi-paragraph "All data will be regularly backed up and backup copies stored both on and off site. Paper records will be archived in locked cabinets. These cabinets will have limited access with prior authorisation. All data will be partially- anonymized prior to presentation or publication of any results".

**Response: **We have made some changes in the way this is reported. The final sentence have been retained "All electronic data will be regularly backed up with copies stored both on and off site. Paper records will be archived in locked cabinets which have limited access with prior authorisation".

 3. Some abbreviations are missing, e.g. FBC and POC.

**Response: **Thank you we have added these.

 4. Clinical assessment. Line no5 suggest to change word "monitoring" to "monitored".

**Response: **Thank you. Changed.

# Reviewer report: Reviewer response for version 1

**DOI:** 10.21956/wellcomeopenres.22271.r71475

**Published:** 2024-01-02

**Authors:** Mei Li

**Affiliations:** 1 National Institute of Parasitic Diseases, Chinese Center for Disease Control and Prevention, Shanghai, China

It is the first time to know such interesting practice of publishing article. Only the STUDY PROTOCOL is available.

 Basing on positive results in adult, this study aims to assess safety, dosing, feasibility of sevuparin as an adjuvant therapy in children with severe malaria complicated by lactic acidosis (> 2mmol/l). In my opinion, the rationale for, and objectives of, the study is clearly described, the study design appropriate for the research question, sufficient details of the methods provided to allow replication by others, and the datasets are clearly presented in a useable and accessible format in the article.

 However, there is an uncertain point.  Is it the most suitable means to measure parasite biomass using histidine-rich protein2 (pfHRP2)? Is there evidence to support this usage? PfHRP2 might persist for a long time in successfully treated patients  who had been infected by falciparum parasites, especially in children [ Dalrymple U， Arambepola R， Gething PW，et al. How long do rapid diagnostic tests remain positive after anti-malarial treatment? Malar J, 2018,17:228.]. Sevuparin, as a potential adjunctive treatment, will be used together with anti-malarial drugs. The change of parasite biomass or parasitemia is very important to following the patients’ condition. So, parasite density is suggested to count in thin films or thick film (≤40,000 parasites/μL blood) except of pfHRP2 test.

Is the study design appropriate for the research question?

Yes

Is the rationale for, and objectives of, the study clearly described?

Yes

Are sufficient details of the methods provided to allow replication by others?

Yes

Are the datasets clearly presented in a useable and accessible format?

Yes

Reviewer Expertise:

I mainly work on malaria diagnosis and control, and have taken part in the TES for anti-malaria drugs.

I confirm that I have read this submission and believe that I have an appropriate level of expertise to confirm that it is of an acceptable scientific standard.

# Response

**Published:** 2024-08-02

**Authors:** Kathryn Maitland

**Affiliations:** KEMRI-Wellcome Trust Research Programme, Kenya

We thank the reviewer for a positive appraisal the the trial protocol. With regards to the question on measurement of parasite biomass using PfHRP2 there is a substantial literature on its superiority in estimating true biomass rather the parasitaemia. It was first described by the coauthors of this trial protocol. Of specific relevance to severe malaria, which results from extensive sequestration of parasitised erythrocytes, sequestered parasites secrete PfHRP2 into the plasma which is liberated at schizont rupture. The plasma concentration of PfHRP2 provide a better estimate for the patient's total parasite biomass, rather than peripheral parasitaemia (which assesses the less mature and pathogenic circulating stages ( https://doi.org/10.1371/journal.pmed.0020204). In addition, PfHRP levels more accurately predict children who have true severe malaria from those with incidental parasitaemia, and is a better indicator of prognosis rather than parasitaemia ( https://doi.org/10.1093/infdis/jis675). We understand that low levels of HRP2 can persist even after clearance of malaria parasites. PfHRP in our study is being used to stratify risk at admission in children administered with severe and complicated disease.
